# Neuronal network analysis during short time transcranial direct current stimulation with permanent EEG measurement in early childhood: a feasibility study

**DOI:** 10.1007/s00702-025-02946-8

**Published:** 2025-05-27

**Authors:** Hannes Brehme, Michael Kölch, Johannes Buchmann, Christoph Berger

**Affiliations:** Department of Psychiatry, Neurology, Psychotherapy and Psychosomatics in Childhood and Adolescence, Rostock University Medical Center, Gehlsheimer Straße 20, 18147 Rostock, Germany

**Keywords:** tDCS, EEG, Childhood, Developing brain, Non-invasive stimulation

## Abstract

Transcranial direct current stimulation (tDCS) as a non-invasive stimulation is still in the experimental stage for many psychiatric disorders even in adults. The use of tDCS provides an opportunity to influence neural networks and their functional connectivity. How tDCS affects cortical networks and how it influences the functional connectivity of the developing brain is largely unknown. The electroencephalography (EEG) derivation, which has a precise temporal resolution, is well established in the clinical routine in childhood and, in combination with tDCS, it will give us more information about the changes in connectivity during stimulation. Furthermore, objective stress markers should be established for non-invasive stimulation at younger ages. In this study, we investigated how well we can measure connectivity changes under tDCS in children aged 4 to 8 using EEG. The exclusion criterion was epilepsy. Anodal stimulation of the left dorsolateral prefrontal cortex was performed. Throughout the stimulation, EEG and ECG measurements were taken. Heart rate variability was calculated from the ECG lead as a stress marker. The combination of tDCS and EEG was applicable and not associated with increased stress levels. It was shown to be technically feasible and to provide information about changes in connectivity. This may allow a more specific and targeted use of tDCS in the developing brain. It would be convenient to use it in children because it does not require pharmacological intervention, which children can tolerate, unlike, for example, fMRI.

## Introduction

Non-invasive neuromodulation methods as transcranial direct current stimulation (tDCS) are safe treatments in adults in different psychiatric disorders (major depressive disorder, schizophrenia, anxiety and obsessive compulsive disorder) (Baeken et al. 2016). But more than 200 years after tDCS was first used on"melancholic"patients (Parent 2004), it remains experimental for many psychiatric disorders and is not yet approved by regulatory agencies such as the FDA.

As reported by Auvichayapat and Auvichayapat (2022), the effects of tDCS on growing brains, as opposed to adults, and the influence of maturation, remain poorly understood. There may be methodological differences between adults and children and adolescents: In adulthood, there are well-established principles of strategic electrode placement (Woods et al. 2016), local neuronal increases and decreases under anodal or cathodal electrodes (Nitsche and Paulus 2000; Stagg et al. 2018), and even long-term effects via NMDA-related cortical long-term potentiation and depression (Nitsche et al. 2006; Stagg et al. 2009; Krause et al. 2013). Data on the effects of tDCS on the developing brain are currently lacking, but the safety of its use in younger age groups has been demonstrated (Muszkat et al. 2016). In some MRI studies, current flow has been used to model the resulting electric field during tDCS (Ciechanski et al. 2018), and the need to adjust stimulation parameters in childhood has been identified. Other previous studies have found age-specific effects of tDCS on cortical excitability in the primary motor cortex (Moliadze et al. 2015). However, the extent to which tDCS affects other regions and changes in connectivity, for example by stimulating the left dorsolateral prefrontal cortex (DLPFC), is not fully understood. In child and adolescent psychiatry, the DLPFC is a target region, for example in attention-deficit/hyperactivity disorder (ADHD) (Westwood et al. 2021). The effect of neuromodulation on the DLPFC is more known in adults (Struckmann et al. 2022), then in developing brain. Most previous studies in children using tDCS for psychiatric (attention deficit/hyperactivity disorder, autism, dyslexia) and neurological (epilepsy, dystonia, cerebral palsy) conditions have to be extrapolated from adult data (Krishnan et al. 2015; Palm et al. 2016; Finisguerra et al. 2019), leading to different conclusions about efficacy in children. There is clearly a need to learn more about the connections in the developing brain. Because of these aspects, the aim of the present study is to investigate connectivity changes under tDCS in early childhood using electroencephalography (EEG), which is well established in childhood and its derivation has a precise temporal resolution, allowing us to see immediate changes even with short stimulation periods.

Furthermore, little is known about the burden on young children caused by such an examination procedure. Therefore as marker for tolerability we choose the Heart rate variability (or variance) (HRV) as hint for the autonomic nervous system tone is an indirect marker for stress level (Berntson et al. 1997; Cygankiewicz and Zareba 2013). The participants are of a certain age, and the questionnaires that have been used are not validated. Therefore, HRV is a pragmatic and objective marker for the responsiveness of the autonomic nervous system. Consequently, HRV can be used as an indirect stress marker.

## Methods

This study was conducted as a single-center study and was registered on DRKS following approval by the ethics committee.

### Participants

Inclusion criteria were age between 4.0 and 8.0 years. Exclusion criteria were epilepsy and epileptic discharges on the participant's EEG. We included eleven children aged between four and eight years, of whom 5 were female and 6 were male. The median age was 5.4 years. The parents of the participants were interviewed about developmental milestones and family history of epilepsy. The children underwent a neurological examination. The clinical impression was that all the children were developing normally for their age. Based on the NIBS Tolerability Score for Adolescents [Appendix A from Zewdie et al. 2020], each child was asked in age-appropriate language after stimulation about side effects such as headache, toothache, itching, burning, visual phenomena, hearing problems, nausea and fatigue. Skin redness was assessed and followed up the next day.

### Data acquisition (EEG and tDCS)

EEG recording was performed by a certified medical technician using the XLTEK EEG system (eeg32u amplifier, Natus Europe GmbH, Planegg, Germany). Continuous EEG was recorded while participants were comfortably seated in a semi-reclined chair with eyes closed. 19 electrodes were placed according to the international 10–20 basic system (Fp1, Fp2, F7, F3, Fz, F4, F8, T3, C3, Cz, C4, T4, T5, P3, Pz, P4, T6, O1 and O2) and referenced to the connected earlobes. The ground electrode was placed between the Fz and Cz electrodes. The placement of two ECG electrodes (Derivation 1) was conducted with the objective of detecting cardio ballistic artefacts and calculating heart rate variability. EEG data were sampled at 1024 Hz. The filter settings during measurement were 0.1 Hz low cutoff and 400 Hz high cutoff. BrainVision Analyzer was used in version 2.3.0.8300. The NeuroConn DC-STIMULATOR PLUS (neurocare group AG, Albert-Einstein-Str. 3, 98683 Ilmenau, Germany) was used for transcranial direct current stimulation (tDCS). The anodal electrode (2.5 cm in diameter) was positioned over the dorsal left prefrontal cortex (DLPC), the cathodal electrode over the right clavicle. 1 mA was applied for two minutes. EEG data were recorded for approximately 10 min before, 2 min during and 10 min after stimulation. Due to artefacts, the ECG was recorded continuously throughout the measurement, so that the determination of heart rate variability is not an additional burden.

### Heart rate variability

Raw data.txt files (time from R to R peak = RR) were exported from the BrainVision Analyzer software version 2.3.0 and then processed using Kubios HRV Premium software version 3.5 (Biosignal Analysis and Medical Imaging Group, Department of Physics, University of Kuopio, Kuopio, Finland). The pre-processing settings were set to the default values, including the RR detrending method, which was left at"smoothness priors"(lambda = 500). The RR series was then corrected using the Kubios HRV Premium"automatic method"(Lipponen and Tarvainen 2019). Data sets with artefacts > 3% were excluded from the analysis. In the time domain, the squared root mean of the sum of the squares of successive normal R-R interval differences (RMSSD) in ms was calculated, and in the frequency domain, the high frequency (HF 0.15–0.4 Hz (Hz)) power was derived. RMSSD reflects the beat-to-beat variance in HR and is an estimate of vagally mediated changes in HRV (Shaffer and Ginsberg 2017). The HF band reflects parasympathetic activity and is called the respiratory band because it corresponds to the HR variations associated with the respiratory cycle (Grossman and Taylor 2007).

### EEG preprocessing pipeline

Further processing was performed using the Brainvision Analyzer (Mesmed GmbH, Gilching, Germany). The EEG data were pre-processed according to the manual of the VIGALL toolbox (https://research.uni-leipzig.de/vigall/), including the following steps Filtering with a Butterworth zero-phase filter (0.5–70 Hz, notch at 50 Hz), creation of 2 s segments, rough artefact screening by visual inspection, performance of independent component analysis (ICA) with the infomax algorithm and exclusion of ICA components reflecting continuous artefacts such as blinks, eye movements and cardioballistic artefacts, and finally marking of remaining artefacts. The EEG data were searched for sleep graphoelements (sleep spindles, K-complexes), but none were identified, which was expected because the assistant was constantly monitoring the EEG recording during acquisition and prevented the subject from directly falling asleep.

### Analysis of cortical source of EEG rhythms (LORETA)

We used low resolution electromagnetic source tomography (LORETA) as provided at http://www.unizh.ch/keyinst/NewLORETA/LORETA01.htm for the estimation of cortical sources of scalp EEG power density (Pascual-Marqui et al. 1994, 2002). LORETA solutions consisted of 7 mm isometric voxel current density values able to predict EEG spectral power density at scalp electrodes. Taken into account the low number of scalp electrodes we averaged the LORETA solutions for cortical larger regions of interest (ROI) such as frontal, central, parietal, occipital and temporal regions (Table 1 lists the ROIs as Brodmann areas, defined within the LORETA software) as described before (Lizio et al. 2016). The frequency bands of interest were delta (2–4 Hz), theta (4–8 Hz), alpha 1 (8–10.5 Hz), alpha 2 (10.5–13 Hz), beta 1 (13–15 Hz), beta 2 (15–20 Hz) and beta 3 (20–40 Hz).

**Table 1:** The present study employs regions of interest (ROIs) for the estimation of cortical sources of resting state EEG rhythms

Frontal	8, 9, 10, 11, 44, 45, 46, 47
Central	1, 2, 3, 4, 6
Parietal	5, 7, 30, 39, 40, 43
Temporal	20, 21, 22, 37, 38, 41, 42
Occipital	17, 18, 19

The Brodmann areas are utilised in accordance with the definitions provided by the low-resolution brain electromagnetic tomography (LORETA) freeware

### EEG connectivity analysis

We examined the phase synchrony as a measure of connectivity between the EEG channels using the weighted phase lag index (wPLI). The wPLI measures the phase consistency at a specific time and frequency examined across a pair of electrodes or clusters that minimizes activity likely due to volume conduction. WPLI is denoting synchrony between two oscillations as one if the phase angle differences are constant over time and if the signals are fluctuating asynchronous, wPLI will be 0 (for the mathematical details see (Vinck et al. 2011)).

$$wPLI=\left|\frac{\sum_{x=1}^{n}\left|Imag\left[{e}^{i\left({\phi }_{jtf}-{\phi }_{ktf}\right)}\right]\right|sgn\left(Imag\left[{e}^{i\left({\phi }_{jtf}-{\phi }_{ktf}\right)}\right]\right)}{\sum_{x=1}^{n}\left|Imag\left[{e}^{i\left({\phi }_{jtf}-{\phi }_{ktf}\right)}\right]\right|}\right|$$

WPLI was calculated between every pair of electrodes for each 2-s-segment and each frequency separately using the MATLAB scripts from Morales and Bowers (2022)). Furthermore we used a graph analysis approach to characterize the whole brain resting state network interactions (Stam et al. 2014). We calculated a minimum spanning tree (MST) as a representation of the network and from that metrics can be derived to describe the communication flow in the overall network. A network with “Small world” topology is assumed as highly efficient and consist of an optimal combination of high number of local interconnections and short path length (Medaglia et al. 2015). The MST is a sub graph connecting all nodes without loops, based on a matrix of link weights between the nodes. The MST was built by using the Kruskal’s algorithm (Kruskal 1956). This algorithm iteratively selects the links with the lowest weights and adds the link to the tree, if no loops are created. We define a link weight as 1-wPLI and therefore the MST represents the sub-network with maximum connectivity.

To describe the topological properties of the resting state network, we examined the following MST metrics: maximal degree (MD), number of leafs (L), strength (S), diameter (d), closeness (C), betweenness centrality (BC), eccentricity (E), imbalance index (II), kappa (K). Higher values of MD, C, BC and a lower K indicating a higher regional importance of some nodes, which may be considered as hubs. An increase in diameter, means a decrease in global efficiency, whereas a low diameter indicates a more efficient information flow between brain regions. A description of these metrics is provided in Table 2, for further details see also (Stam et al. 2014; Boersma et al. 2013; Tewarie et al. 2014).

**Table 2:** Explanation of abbreviation in the minimum spanning tree (MST) metrics

Symbol	Label	Explanation
MD	Maximal Degree	The maximum number of node connections (= links) of all nodes
L	Number of Leafs	Number of nodes with degree = 1
S	Strength	A summation of all nodal link weights
D	Diameter	Indicating network efficiency and refers to the largest distance (in number of links) between any two nodes and is normalized for the total number of links
C	Closeness	The inverse of the sum of all distances (shortest paths) to other nodes. The closeness of the MST is the maximum closeness of all nodes
BC	Betweenness centrality	Relies on the identification of the number of shortest paths that pass through a node. BC for the whole MST is the maximum of BC of all nodes
E	Eccentricity	The eccentricity is the longest distance between a reference and any other node The eccentricity of the whole MST is the difference between the eccentricity values of the nodes with the largest and smallest eccentricity in the tree
II	Imbalance index	A hierarchical metric that quantifies the trade-off between large scale integration in the MST and the overload of central nodes
K	Kappa	A measure of the broadness of the degree distribution or the heterogeneity of degrees and relates to the spread of information across the tree

### Statistical analyses

Because of the small sample size, HRV and EEG connectivity data (mean wPLI over all electrodes and MST metrics) as well as EEG power density were analyzed for tDCS effects compared to baseline (treatment vs baseline and post treatment vs baseline) using the Wilcoxon test (two sided p values). The significance level was set at 0.05.

## Results

### Participants

No serious adverse effects were observed. Mild adverse effects, including erythema (reported in three children) and pruritus, were documented in nine of the 11 children. The erythema resolved by the following day. No additional adverse effects were identified or reported.

### Heart rate variability

**Fig. 1:**
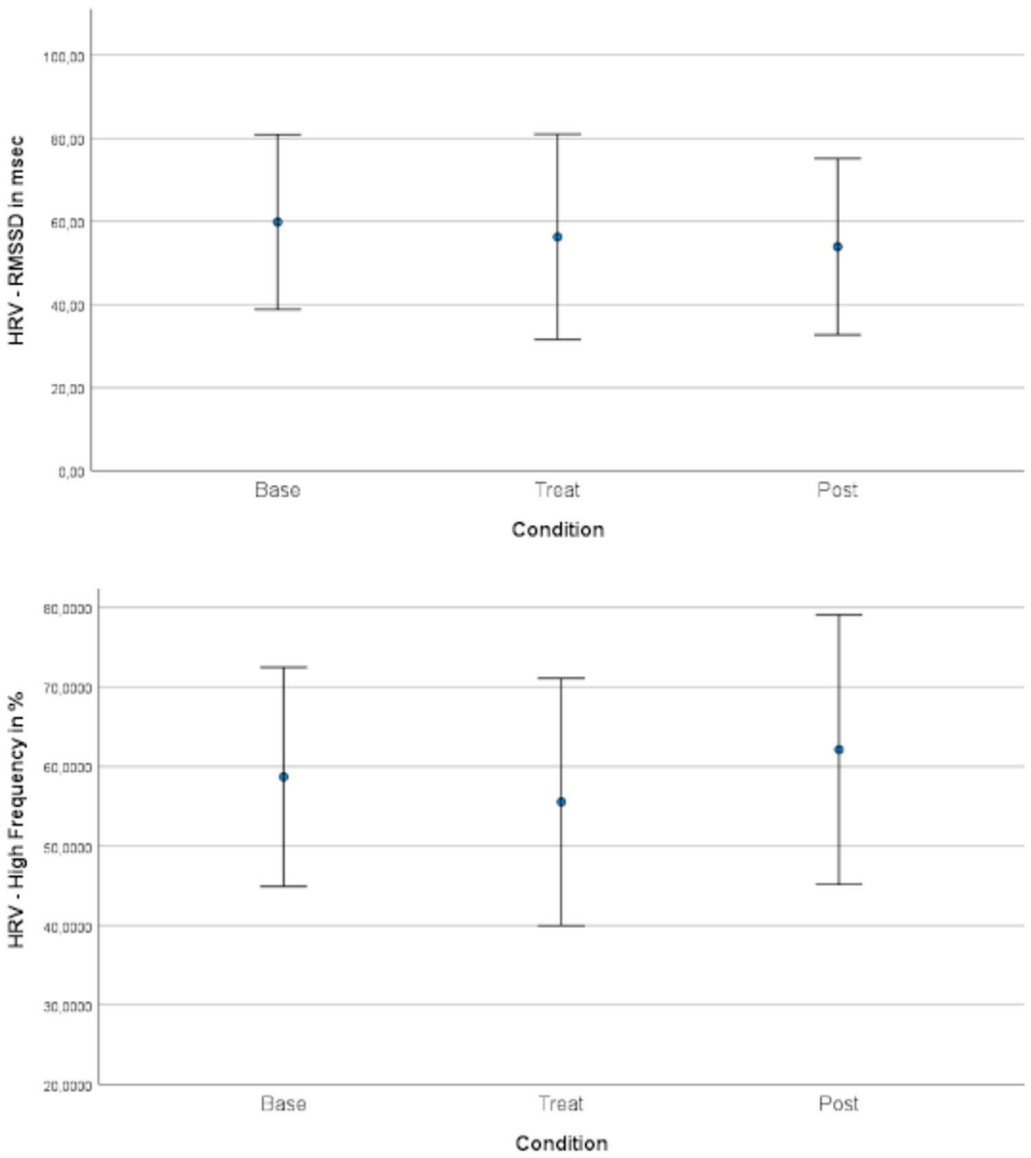
The heart rate variance of the children before (base), during (treat) and after (post) stimulation. There is a statistically significant change for RMSSD comparing post stimulation and baseline, but with small Cohen’s d of 0.14. No significant HF frequency change compared to baseline indicating that the stress levels of the participants remain rather stable throughout the stimulation period. The error bars represent a 95% confidence interval

The Root Mean Square of Successive Differences (RMSSD)) was significantly increased after stimulation (with a small effect size of Cohen’s d = 0.14) but not during stimulation of the relative power of high frequency band activity was not significantly changed (Table 3 and Fig. 1). Taken together, this finding implies that the activation of the vegetative nervous system remained mainly unaltered as a consequence of the stimulation, and thus the stress level remains mainly unaffected.

**Table 3:** Changes in heart rate variability during stimulation and post stimulation compared to baseline

HRV value	Mean (SD)			p value	
	Baseline	Stimulation	Post Stimulation	Stimulation – Baseline	Post – Baseline
RMSSD in ms	60 (29)	56 (34)	54 (30)	0.285	**0.028**
High frequency in %	59 (19)	56 (22)	62 (24)	0.646	0.721

Significant p-values (<0.05) ​​are marked in bold

### Quantitative EEG data

**Fig. 2:**
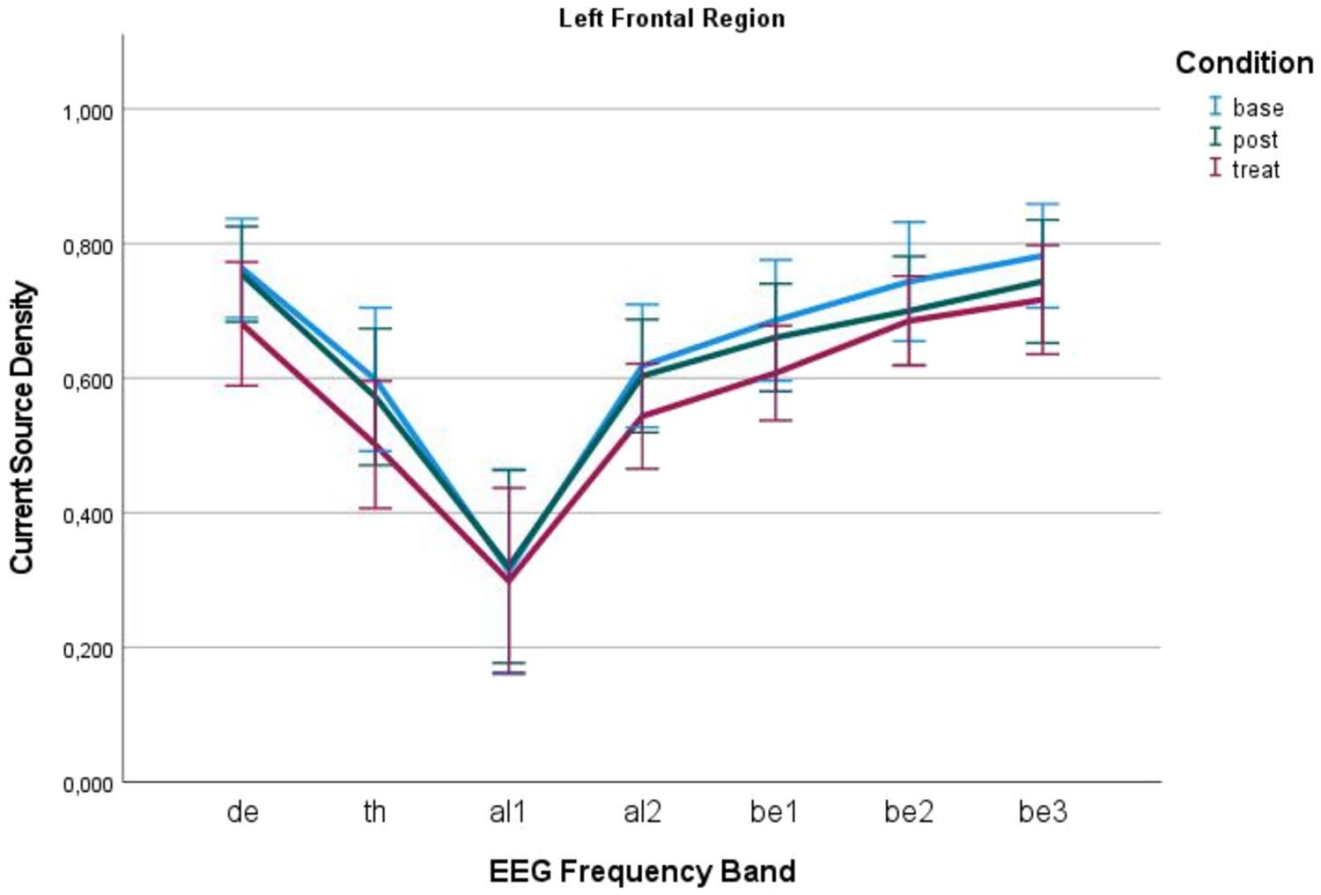
The different frequencies of the EEG in the left frontal region. Post-stimulation, marginal changes were noted in the form of alpha stabilization, though these did not reach statistical significance. The error bars represent a 95% confidence interval. In conclusion, the power analysis did not reveal any significant differences within the stimulated region

**Fig. 3:**
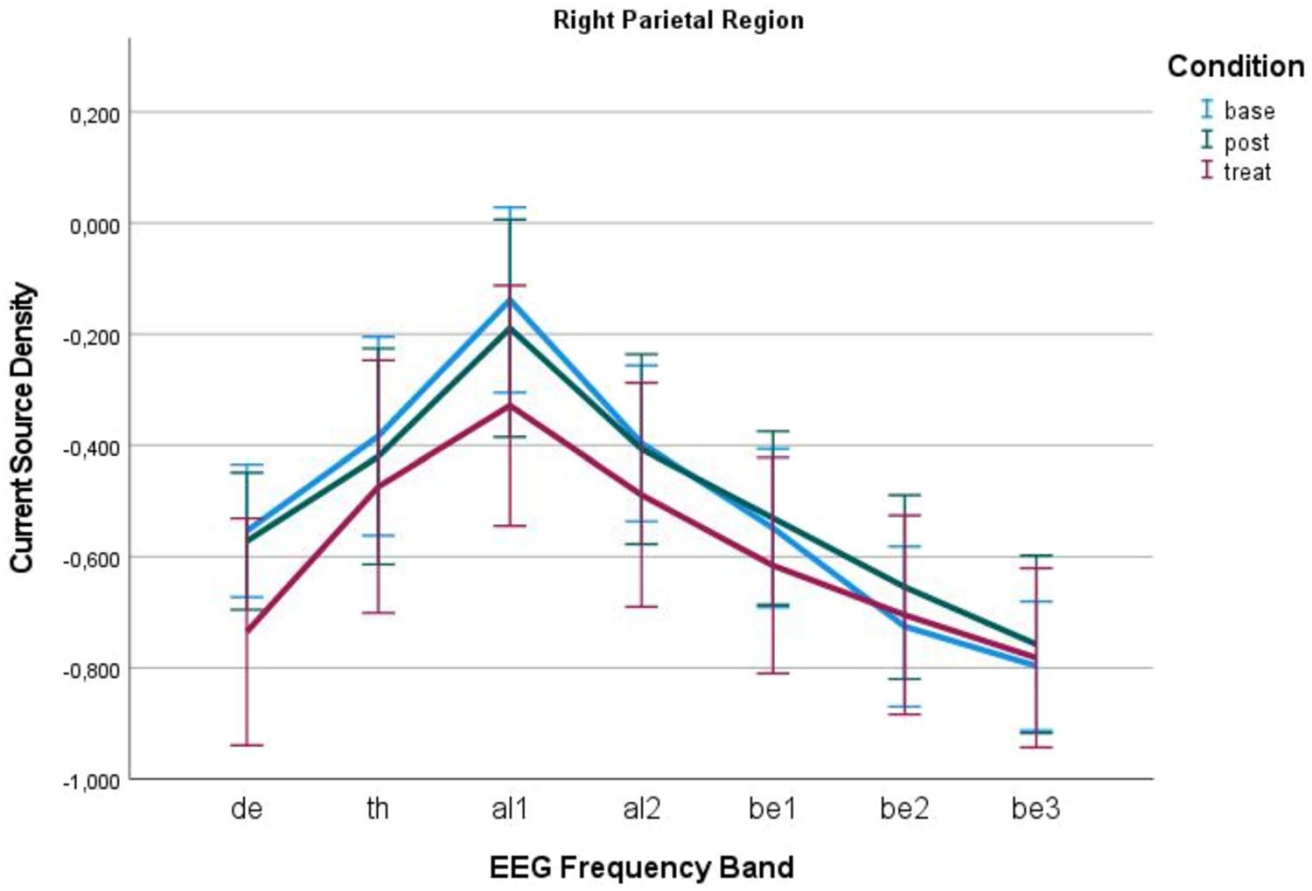
The different frequencies of the EEG in the right posterior region. Following stimulation, the contralateral posterior region appears to exhibit a slight suppression. No lasting effect is observed after two minutes of stimulation. The error bars indicate a 95% confidence interval. In conclusion, the power analysis demonstrates alterations in the contralateral region in response to stimulation

A mere 5% of the EEG data sets was excluded due to the presence of artefacts. Over all, normal cortical structures were observed, exhibiting an alpha peak in the posterior occipital lobe and a predominance of beta rhythm in the frontal lobe. To illustrate, the results of the power analysis in the stimulated left frontal region are presented in Fig. 2. The changes in power of current source density only reached significance from baseline to stimulation and from baseline to post stimulation in the delta band for both hemispheres. Nevertheless, the power changes in the contralateral centroparietal region (Fig. 3) demonstrates a reduction in the alpha band. These results demonstrate that the stimulation exerts not only a regional influence but can also influence all areas in power via connective influences. For all p values see Table 4.

**Table 4:** Changes in power source density from LORETA analysis during stimulation and post stimulation compared to baseline

Test	Hemisphere	Band	p value				
			Frontal	Central	Parietal	Occipital	Temporal
Stimulation—baseline	Left	Delta	0.508	**0.017**	0.445	0.203	0.285
		Theta	0.386	0.386	0.203	0.386	0.508
		Alpha1	0.646	0.575	0.508	0.646	0.508
		Alpha2	0.799	0.646	0.203	0.285	0.575
		Beta1	0.445	0.203	0.139	0.575	0.074
		Beta2	0.445	0.203	0.878	0.959	0.074
		Beta3	0.878	0.959	0.799	0.721	0.203
	Right	Delta	0.241	**0.028**	0.139	0.508	0.721
		Theta	0.575	0.959	0.139	0.445	0.878
		Alpha1	0.878	0.646	0.508	0.575	0.445
		Alpha2	0.799	0.114	0.386	0.799	0.646
		Beta1	0.508	0.114	0.139	0.508	0.646
		Beta2	0.285	0.139	0.285	0.799	0.333
		Beta3	0.575	0.799	0.508	0.721	0.878
Post—baseline	Left	Delta	0.508	**0.017**	0.445	0.203	0.285
		Theta	0.386	0.386	0.203	0.386	0.508
		Alpha1	0.646	0.575	0.508	0.646	0.508
		Alpha2	0.799	0.646	0.203	0.285	0.575
		Beta1	0.445	0.203	0.139	0.575	0.074
		Beta2	0.445	0.203	0.878	0.959	0.074
		Beta3	0.878	0.959	0.799	0.721	0.203
	Right	Delta	0.241	**0.028**	0.139	0.508	0.721
		Theta	0.575	0.959	0.139	0.445	0.878
		Alpha1	0.878	0.646	0.508	0.575	0.445
		Alpha2	0.799	0.114	0.386	0.799	0.646
		Beta1	0.508	0.114	0.139	0.508	0.646
		Beta2	0.285	0.139	0.285	0.799	0.333
		Beta3	0.575	0.799	0.508	0.721	0.878

P values from Wilcoxon paired test

### EEG connectivity analysis

**Fig. 4:**
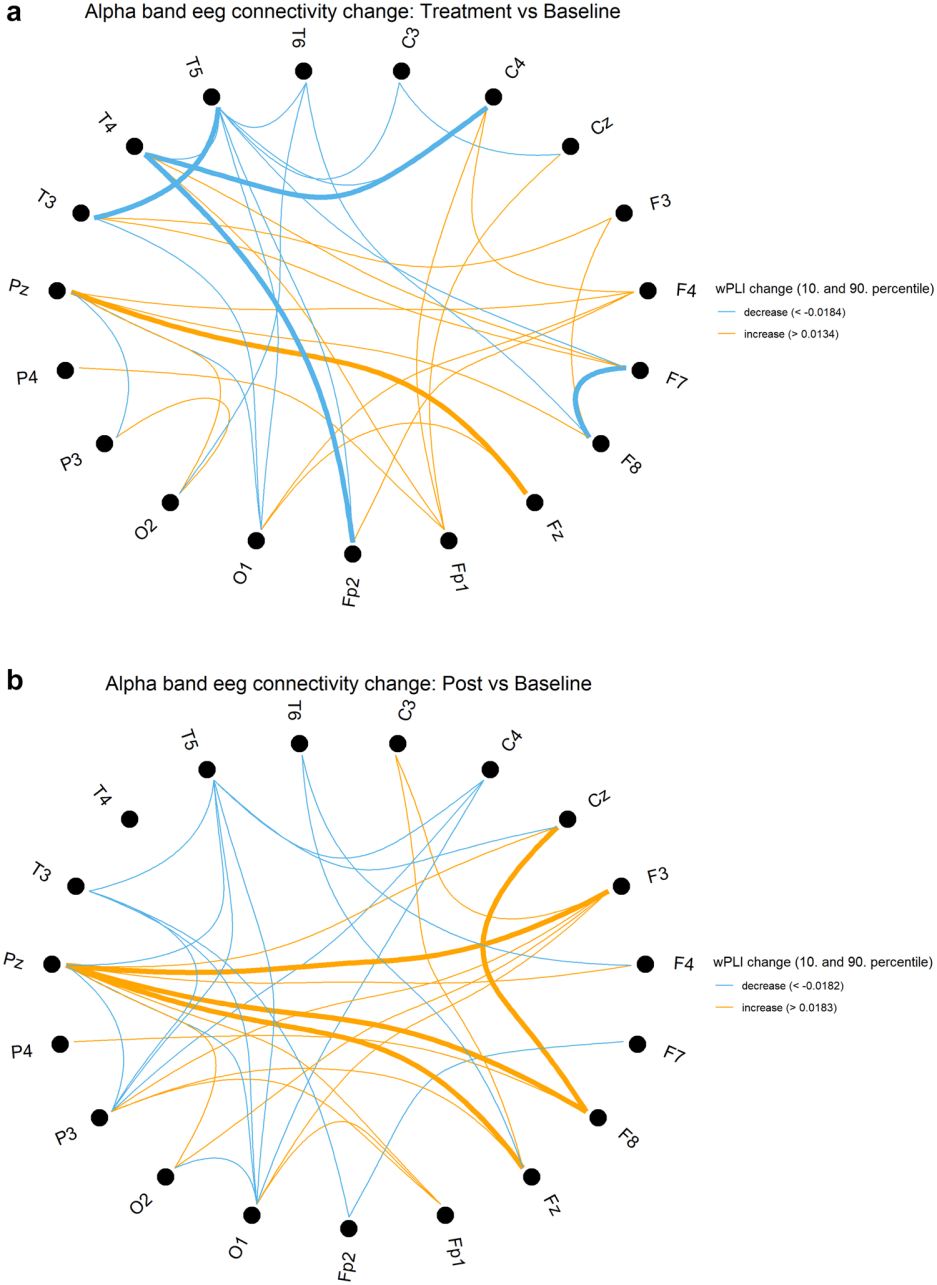
Changes in functional connectivity within the alpha spectrum, using wPLI metrics. Figures 4 & 5 compare connectivity between electrodes during stimulation (**a**) and post-stimulation (**b**) to the resting-state EEG recorded prior to stimulation. The figure 4 highlights changes in the alpha band. The results show a significant increase in interregional fronto-parietal network connectivity, particularly during the post-stimulation state. The 10 th percentile (light blue) and 90 th percentile (orange) of the wPLI value distributions across all channel pairs are displayed. Bold lines indicate connections with significant changes (p value < 0.05, uncorrected)

**Fig. 5:**
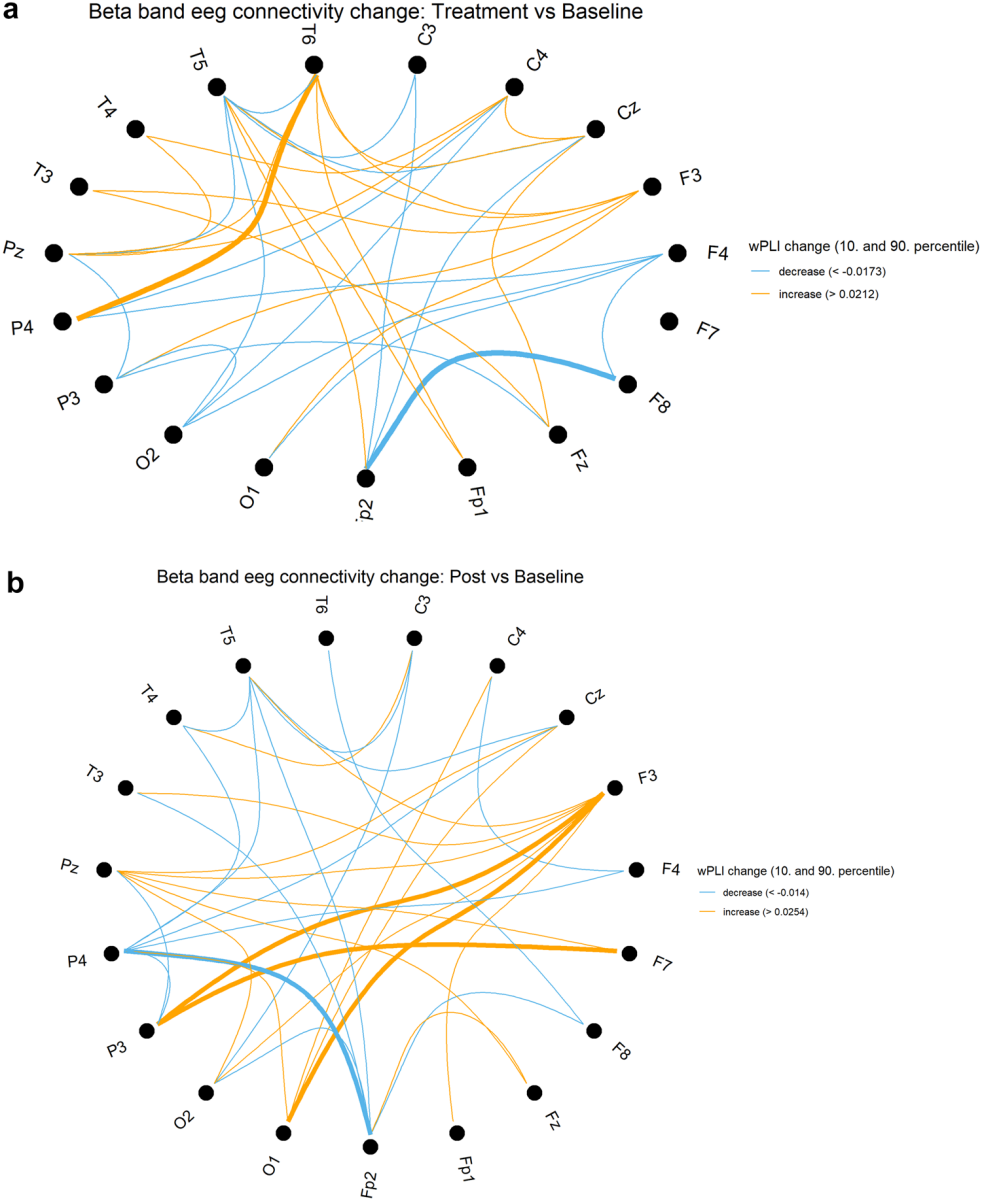
Changes in functional connectivity within the beta spectrum, using wPLI metrics. Figures 4 & 5 compare connectivity between electrodes during stimulation (**a**) and post-stimulation (**b**) to the resting-state EEG recorded prior to stimulation. This figure focuses on changes in the beta band. The results show a significant increase in interregional fronto-parietal network connectivity, particularly during the post-stimulation state. The 10 th percentile (light blue) and 90 th percentile (orange) of the wPLI value distributions across all channel pairs are displayed. Bold lines indicate connections with significant changes (p value < 0.05, uncorrected)

The analysis of functional connectivity across all electrode pairs, using wPLI metrics, revealed notable findings. There was a significant strengthening of fronto–temporo–parietal network connectivity in both the left and right hemispheres, particularly during the post-stimulation state within the alpha and beta bands (Tables 5 and 6; Figs. 4 and 5, respectively).

**Table 5:** All connections with functional connectivity metrics showing significant but unadjusted p values during stimulation compared to baseline

wPLI changes during stimulation compared to baseline				
Channel 1	Channel 2	Frequency band	wPLI change	p value
O1	T6	Delta	– 0.027	0.037
P3	T4	Delta	– 0.016	0.049
C3	F3	Delta	0.011	0.028
F4	T4	Delta	0.021	0.037
Fp2	P3	Delta	0.022	0.037
Cz	T5	Delta	0.027	0.027
F3	F7	Delta	0.030	0.002
F3	T6	Delta	0.031	0.037
Cz	Fz	Delta	0.031	0.037
T4	T6	Delta	0.032	0.004
F7	T6	Delta	0.034	0.020
Fp2	T3	Delta	0.041	0.037
Cz	O2	Theta	– 0.042	0.037
Fp2	O1	Theta	– 0.018	0.049
F3	Fz	Theta	– 0.013	0.006
C4	T5	Theta	0.013	0.014
Fp2	T4	Alpha	– 0.027	0.010
T3	T5	Alpha	– 0.025	0.027
C4	T4	Alpha	– 0.025	0.049
F7	F8	Alpha	– 0.016	0.020
Fz	Pz	Alpha	0.017	0.037
F8	Fp2	Beta	– 0.023	0.014
P4	T6	Beta	0.013	0.004

Note, that these connections were not significant anymore after applying Benjamini–Hochberg procedure to control for false discovery rate

**Table 6:** All connections with functional connectivity metrics showing significant but unadjusted p values during post – stimulation compared to baseline

wPLI changes post stimulation compared to baseline				
Channel 1	Channel 2	Frequency band	wPLI change	p value
P3	P4	Delta	– 0.034	0.037
P4	Pz	Delta	– 0.028	0.049
P4	T6	Delta	– 0.025	0.020
Pz	T6	Delta	– 0.021	0.014
F4	Pz	Delta	– 0.017	0.049
F4	T6	Delta	– 0.017	0.049
O1	Pz	Delta	– 0.016	0.049
F4	O1	Delta	– 0.005	0.049
Cz	T5	Delta	0.014	0.010
C3	Cz	Delta	0.019	0.010
F3	T5	Delta	0.025	0.049
Fp2	O1	Theta	– 0.033	0.010
O1	O2	Theta	– 0.025	0.002
Fp2	P3	Theta	– 0.024	0.020
O2	P3	Theta	– 0.017	0.049
O1	P3	Theta	– 0.010	0.027
C4	O1	Theta	– 0.010	0.049
F4	T3	Theta	0.013	0.049
C3	F3	Theta	0.016	0.037
F3	P3	Theta	0.016	0.049
Fp1	P4	Theta	0.020	0.027
F4	Fp1	Theta	0.024	0.020
F4	Fz	Theta	0.029	0.020
F3	Pz	Alpha	0.029	0.020
Fz	Pz	Alpha	0.030	0.014
Cz	F8	Alpha	0.031	0.037
F8	Pz	Alpha	0.044	0.027
Fp2	P4	Beta	– 0.016	0.025
F7	P3	Beta	0.012	0.049
F3	P3	Beta	0.015	0.049
F3	O1	Beta	0.017	0.010

Note, that these connections were not significant anymore after applying Benjamini–Hochberg procedure to control for false discovery rate

In the alpha band, significant increases in wPLI were observed during stimulation between Fz and Pz, and post-stimulation between F3, F8, Fz, and Pz, as well as between F8 and Cz, compared to baseline. Conversely, during stimulation, a significant decrease in wPLI was noted primarily in temporal and frontal connections, including T3–T5, T4–Fp2, C4, and F7–F8.

In the beta band, wPLI significantly increased during stimulation between T6 and P4 but decreased between Fp2 and F8. Post-stimulation, significant increases were observed between F3–P3, O1, and F7–P3, while a decrease was noted between P4 and Fp2, compared to baseline.

The reported p-values are uncorrected. Notably, wPLI consistently increased during or after stimulation in beta and alpha band for connections involving F3, regardless of statistical significance. For detailed wPLI changes and the statistical parameters from the Wilcoxon test refer Tables 5 and 6.

**Fig. 6:**
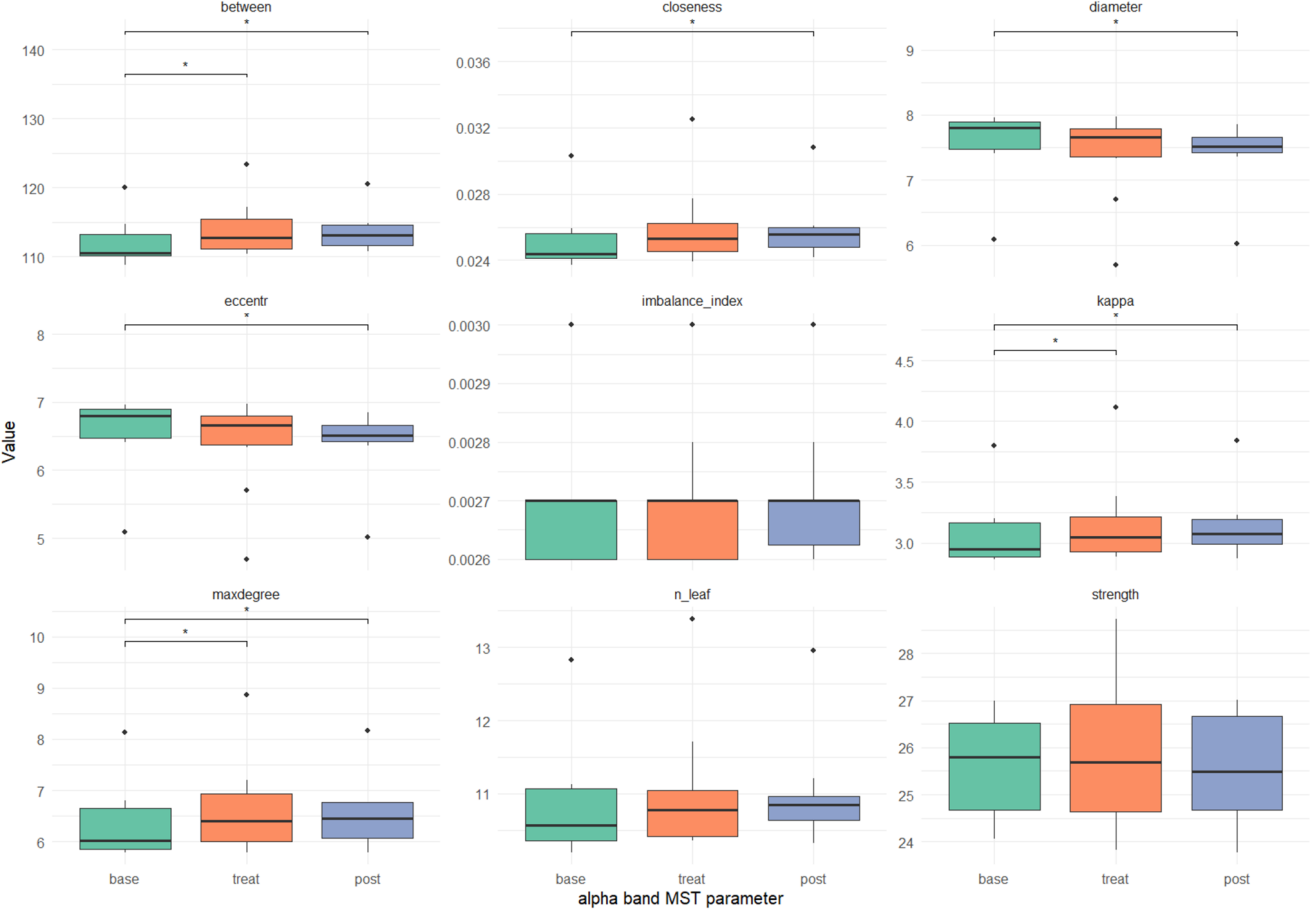
The data obtained from the MST analyses conducted in the alpha spectrum. The significant changes are marked with a *. The error bars indicate a 95% confidence interval. For meaning of the abbreviations please refer to Table 2

Additionally, global organization of functional EEG connectivity was analyzed using the graph theoretical approach MST. Here, significant alterations can be seen only in the alpha band for the MST parameter betweenness, centrality kappa and maximal degree during stimulation and in the MST parameter betweenness, centrality, closeness, excentricity, kappa, maximal degree after treatment. For a complete list of MST values refer to Table 7, Fig. 6 is representing the MST values in Alpha band. Again, the p-values reported are uncorrected.

**Table 7:** Analysis from graphical representation of connectivity measures, minimum spanning tree (MST)

Band	MST Parameter	Mean (SD)			p value	
		Baseline	Treatment	Post Treat	Treatment – Baseline	Post – Baseline
Alpha	Between	112.11 (3.32)	113.95 (4.04)	113.56 (2.88)	**0.011**	**0.014**
	Closeness	0.0252 (0.0019)	0.026 (0.0025)	0.0258 (0.0018)	0.053	**0.009**
	Diameter	7.57 (0.56)	7.39 (0.7)	7.41 (0.51)	0.103	**0.008**
	Eccentricity	6.57 (0.56)	6.39 (0.7)	6.41 (0.51)	0.103	**0.008**
	Imbalance index	0.0026 (1.2e – 04)	0.0027 (1.3e – 04)	0.0027 (1.2e – 04)	1.000	0.571
	Kappa	3.07 (0.29)	3.16 (0.37)	3.14 (0.27)	**0.041**	**0.014**
	Maxdegree	6.36 (0.74)	6.63 (0.92)	6.53 (0.68)	**0.025**	**0.019**
	N leaf	10.85 (0.78)	11.03 (0.93)	11 (0.73)	0.308	0.053
	Strength	25.59 (1.09)	25.82 (1.55)	25.62 (1.15)	0.683	0.760
Beta	Between	113.49 (4.49)	113.92 (4.23)	113.84 (4.07)	0.919	0.415
	Closeness	0.026 (0.003)	0.026 (0.003)	0.026 (0.002)	0.169	0.476
	Diameter	7.35 (0.74)	7.26 (0.73)	7.34 (0.71)	0.185	0.610
	Eccentricity	6.35 (0.74)	6.26 (0.73)	6.34 (0.71)	0.185	0.610
	Imbalance index	0.0027 (1.5e – 04)	0.0027 (1.5e – 04)	0.0027 (1.5e – 04)	0.713	1.000
	Kappa	3.14 (0.44)	3.21 (0.43)	3.16 (0.4)	0.083	0.541
	Maxdegree	6.51 (1.08)	6.68 (1.02)	6.57 (0.96)	0.083	0.476
	N leaf	11.03 (1.06)	11.18 (1.04)	11.07 (0.99)	0.154	0.683
	Strength	30.05 (0.61)	30.25 (0.79)	30.04 (0.61)	0.541	0.541
Delta	Between	112.91 (2.52)	113.63 (2.59)	112.69 (2.47)	0.359	0.919
	Closeness	0.025 (0.0015)	0.026 (0.0014)	0.025 (0.0014)	0.192	0.326
	Diameter	7.48 (0.44)	7.34 (0.42)	7.53 (0.43)	0.221	0.554
	Eccentricity	6.48 (0.44)	6.34 (0.42)	6.53 (0.43)	0.221	0.554
	Imbalance index	0.0027 (8.8e – 05)	0.0027 (9.7e – 05)	0.0027 (8.2e – 05)	0.161	0.766
	Kappa	3.11 (0.23)	3.19 (0.21)	3.1 (0.19)	0.154	1.000
	Maxdegree	6.45 (0.57)	6.66 (0.54)	6.45 (0.48)	0.185	1.000
	N leaf	10.95 (0.61)	11.19 (0.58)	10.92 (0.55)	0.083	0.838
	Strength	20.02 (0.18)	20.01 (0.19)	20.01 (0.33)	0.683	0.919
Theta	Between	112.87 (3.36)	113.03 (3.34)	113.17 (3.19)	0.838	0.760
	Closeness	0.025 (0.002)	0.026 (0.002)	0.026 (0.0021)	0.507	0.760
	Diameter	7.47 (0.58)	7.3 (0.53)	7.49 (0.57)	0.126	0.683
	Eccentricity	6.47 (0.58)	6.3 (0.53)	6.49 (0.57)	0.126	0.683
	Imbalance index	0.0027 (1.2e – 04)	0.0027 (1.0e – 04)	0.0027 (1.2e – 04)	0.234	0.773
	Kappa	3.1 (0.29)	3.12 (0.26)	3.09 (0.3)	0.919	0.610
	Maxdegree	6.43 (0.71)	6.49 (0.67)	6.41 (0.71)	0.683	0.683
	N leaf	10.94 (0.76)	11.1 (0.73)	10.9 (0.78)	0.221	0.610
	Strength	23.75 (0.41)	24.05 (0.58)	23.88 (0.82)	0.126	1.000

Significant p values are shown in bold. Notably all significant changes of MST metrics occurred in the alpha band

## Discussion

The objective of this study was to ascertain the extent to which the combination of electroencephalography (EEG) and transcranial direct current stimulation (tDCS) allows for the formulation of statements about connectivity. Initially, we demonstrated that these measurements are both feasible and well tolerated (3.1 and 3.2). There was no increase in heart rate variability during stimulation, and the incidence of mild side effects was low, with no serious adverse events. Currently, HRV is an adequate and objective marker of stress (Berntson et al. 1997; Cygankiewicz and Zareba 2013) in the absence of validated questionnaires in this age group.

The results indicated that tDCS did not significantly influence the power of the EEG area stimulated (DLPFC). However, it was observed that the connections of both frontal areas were strengthened even in short-time stimulation. There is evidence that the connectivity of this region changes in adulthood, for example in depression (Richieri et al. 2018) and anorexia and bulimia nervosa (Frank et al. 2016). It is notable that both of these conditions occur in childhood and adolescence.

A limitation of the study is that only a small number of healthy children were selected for the study, and the stimulation times were kept relatively brief. This was done for ethical reasons, given the lack of experience with healthy individuals of that age (see (Auvichayapat and Auvichayapat 2022)). Due to the restlessness of movement at this age, it is unavoidable that artefacts will be present in the EEG, and that not all changes can be reliably attributed to the stimulation. However, the high rate of evaluable EEG (see Chapter 3.3) demonstrates that our setting was sufficiently uneventful to allow for the lowest possible variance. It should also be noted that not all measurements were taken at the same time, and fluctuations in vigilance are an inevitable consequence of this. Only a limited number of assertions can be made regarding the generalisability of the findings to larger cohorts. To date, the majority of inferences regarding connectivity have been drawn from fMRI data and computational extrapolation (Richieri et al. 2018; Frank et al. 2016; Park and Friston 2013), particularly in children would be favourable to use. However, there are other studies (Brehme et al. 2025) that examine alterations in EEG following tDCS in children diagnosed with autism. In contrast to the present study, the EEG was recorded before and after several days of stimulation, which limits the extent to which the data can be compared. It is important to emphasise that, although the initial results of Nitsche and Paulus (2000) suggest that stimulation should be for a minimum of three minutes in adults, our study demonstrates that two minutes of stimulation is sufficient to achieve improved connectivity. Consequently, our study supports the hypothesis that the developing brain should be assessed differently (Auvichayapat and Auvichayapat 2022; Brehme et al. 2025) and, if necessary, that EEGs should be performed more frequently than after a completed protocol (Kang et al. 2018).

This study is exploratory in nature and seeks to identify potential trends and patterns rather than to confirm specific hypotheses. Exploratory studies aim to generate hypotheses for future research rather than provide definitive conclusions. Applying stringent corrections for multiple comparisons in an exploratory framework would limit the ability to observe and report preliminary findings that might guide subsequent, more focused investigations. Therefore, we decide not to apply correction for multiple comparism, like the Benjamini–Hochberg (BH) correction in this study. The BH correction is designed to control the false discovery rate (FDR) across a large number of tests, but in small sample sizes, it tends to be overly conservative, leading to the rejection of true signals. In this context, applying BH would likely result in no significant findings surviving the correction, rendering the data uninformative.

## Conclusion

As a non-invasive method, tDCS represents a potential adjunctive treatment in child and adolescent psychiatry. Heart rate variability (HRV) appears to be an appropriate and objective indicator of stress during stimulation. The combination with electroencephalography (EEG) offers a means of enhancing our understanding of the connections within the developing brain, which may ultimately lead to enhanced efficacy of neurostimulation. Furthermore, insights may be gained into the alterations in connectivity during tDCS and the susceptibility of the developing brain, which may assist in enhancing the efficacy of this approach. In contrast to the adult brain, shorter periods of stimulation may be sufficient.

Given the small sample size, the exploratory nature of the study, and the need to balance Type I and Type II errors, we opted not to apply the Benjamini–Hochberg correction. Instead, we focus on identifying potential trends that could inform future, more robustly powered studies. This approach aligns with the goals of exploratory research, which prioritize hypothesis generation over definitive conclusions.
